# Supplementary material for: A Retrospective Assessment of Neuropathic Pain in Response to Intraneural Facilitation® Therapy and Neurovascular Index-Guided Food Elimination
Source: Biomedicines. 2025 Mar 11;13(3):688. doi: 10.3390/biomedicines13030688 (PMC11940592; doi:10.3390/biomedicines13030688)

Supplemental Figure S1. The First Hold

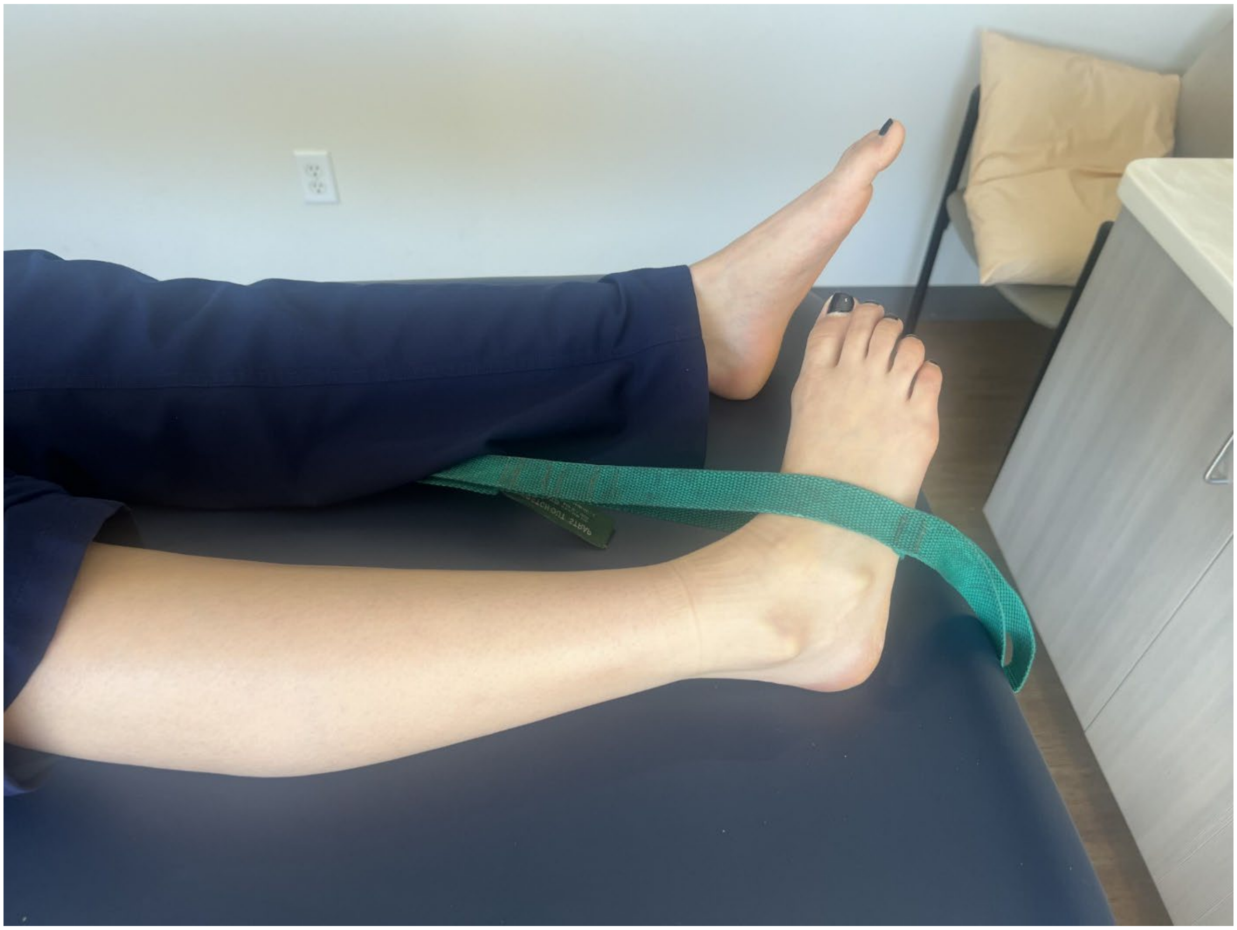

Supplemental Figure S2. The Second Hold

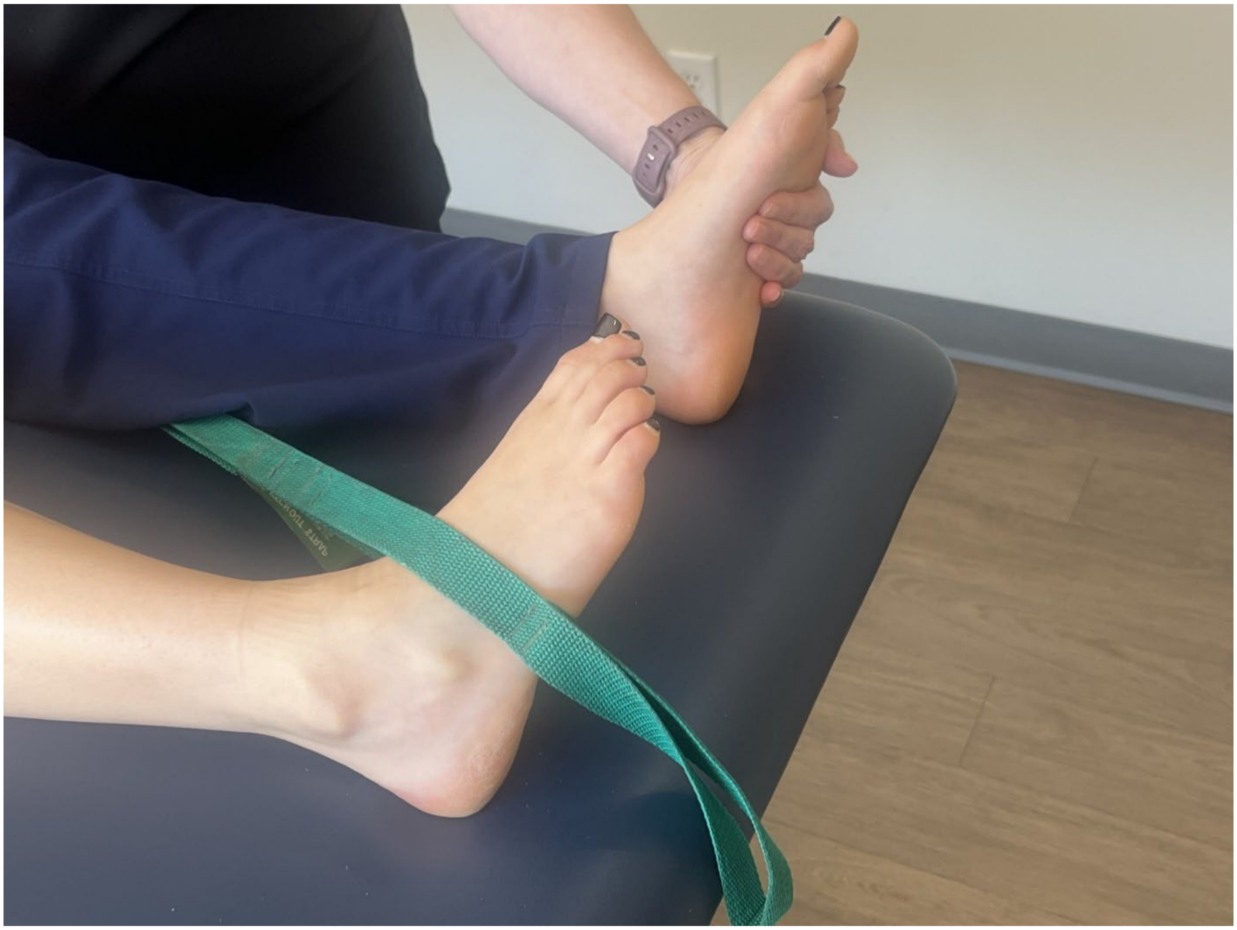

Supplemental Figure S3. The Third Hold

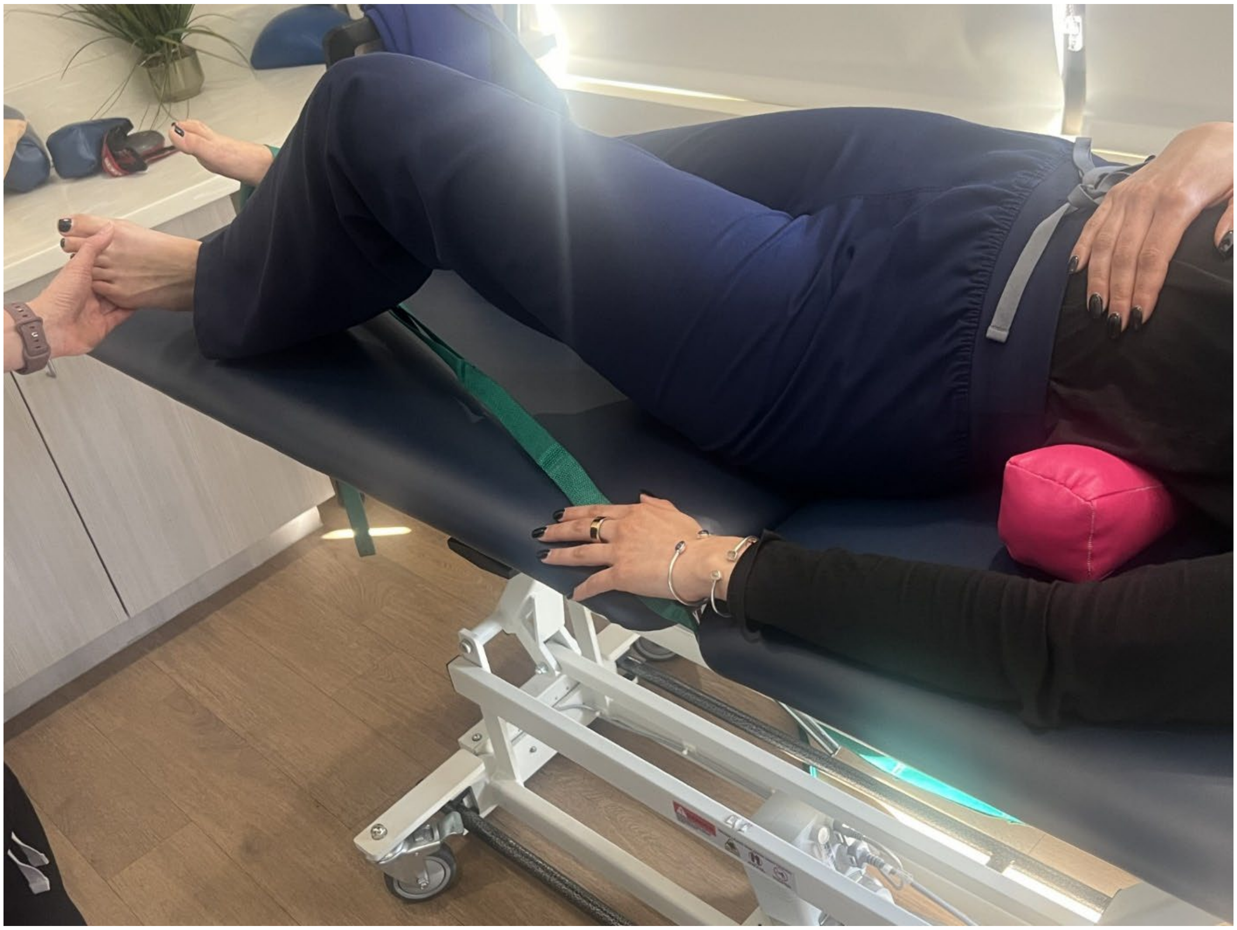

Supplement: Supplementary file 1 [file biomedicines-13-00688-s001.zip › biomedicines-3467367-supplementary.pdf]
